# Demographic Forecasts Using the Game Theory

**DOI:** 10.3390/ijerph16081400

**Published:** 2019-04-18

**Authors:** Marek Ogryzek, Krzysztof Rząsa, Edita Šarkienė

**Affiliations:** 1 Faculty of Geodesy, Geospatial and Civil Engineering, Institute of Geography and Land Management, University of Warmia and Mazury, 15 Prawochenskiego Street, 10-720 Olsztyn, Poland; krzysztof.rzasa@uwm.edu.pl; 2 Department of Roads, Faculty of Environmental Engineering, Vilnius Gediminas Technical University, Sauletekio al. 11, LT-10223 Vilnius, Lithuania; edita.sarkiene@vgtu.lt

**Keywords:** urban design planning, urban development, demographic forecasts, game theory, land management

## Abstract

This paper offers certain predictions concerning the demographic population of the cities Vilnius and Olsztyn. The authors used a method of analyzing and synthesizing data sources, and comparing the actual data with the forecast between the years 1997–2014. Each prediction was prepared in connection with its use in various areas of life, particularly for all studies involving spatial planning. The data collected on the basis of the forecasts were used by spatial planners to devise strategies for local development at the city, municipality, and provincial levels. In this sense, they created basic documents for the sustainable planning of space. The process of forecasting is a difficult and complex issue, and its accuracy determines both the choice of methods and the quality of the output. Our study sets out predictions concerning the demographic processes over the coming years in the two cities mentioned. Given that all long-range forecasts are characterized by high risk, especially taking into account the unstable political situation in Europe, the steadily deteriorating situation in the labor market and rising social discontent are of relevance, as they are causing the ongoing dynamics of the population to change, making statistical errors more likely and more serious. This has meant that organizations like Poland’s Central Statistical Office, Eurostat, and the United Nations have to adjust their demographic projections at least every two years, and the methods for making demographic forecasts which are used by governmental institutions have proven to be less than satisfactory. The main purpose of the article, therefore, is to present the authors’ method of making demographic projections by using elements of game theory. The results obtained in this method were compared with the results of the forecasting methods currently used by the governments of Poland and Lithuania. The developed method, based on the same input data and analogous coefficients, brings more probable results.

## 1. Introduction

Defining the concept of locating the objects on the earth’s surface is one of the basic tasks of Urban Planning (Land Management). This is accomplished by implementing geodata into already existing or created spatial information systems supporting the planning processes. Diagnosing the current socio-economic situation and the conditions of urban development leads to the determination of changes in the projected factors (development poles), on the basis of which directions of spatial development and spatial policies in the area are determined. One of these poles of development is the forecast of demographic growth in the study area. Based on reasonable predictions about demographic development, urban planners can better oversee the necessary conditions of providing basic human needs, as well as deploying investments. According to [1], predictions of the Lithuanian population pose a particular challenge. The main difficulty in predicting the long-term population is the socio-economic situation of the country, as well as political events, such as accession to the EU. Matysiak et al. [2] confirmed similar difficulties in forecasting Polish demographic developments. In 1990, Poland experienced a fundamental change in the system, and Lithuania announced its independence. In 2004, the two countries joined the European Union. These are similar situations, and the political, economic, and social changes that accompanied the transformation have had a great impact on the demographic structure of both countries. When comparing the actual situation in the Polish and Lithuanian demographies, there was a decrease in population in both cases—by −37.6 per 1000 inhabitants in Poland, and −28.4 per 1000 inhabitants in Lithuania (Eurostat, 2014). Negative population growth was due to emigration to Western Europe to find jobs and improve living conditions. International migration is one of the effects of Polish and Lithuanian accession to the EU, and is expected to increase in the future.

According to Holzer [3], the main problem in population forecasting is the period of forecasted time and the evaluation system of the parameter’s distribution in space. Changes in the shape and structure of the population are long-term processes; therefore, the long-term forecast does not explicitly provide real value, but only determines the potential development of the study area. Already in 1985, Alho and Spencer [4] pointed out that, "It is possible to express the uncertainty of the jump-off population in probabilistic terms. This has not been done, because the current version of the PEP does not support this source of uncertainty”.

Forecasting the population is an important element of urban planning practices and land use. When managing space, it is important to include, among other things, urban greenspace, as it has a positive impact on reducing psychological stress among adolescents [5,6,7]. The uncontrolled development of urbanization has a negative impact on the urban population [8,9,10] (Nisbet et al., 2009, Cox et al., 2018, Barton et al., 2010). Larger population growth creates three solution models. The first one is the construction of housing estates with high multi-family housing, using a smaller area and limiting the decrease in the rural areas. The second is increasing cities at the expense of rural areas, using single-family houses. The second model can be considered better in the context of urban development, due to the beneficial effects on the health of the population thanks to home gardens and rural-urban green spaces [11,12,13]. Another important design element is the distance a person has to overcome to arrive at a natural area. The availability and ecological indicators are equally important [14,15]. It is therefore reasonable to combine accessibility to services and green space planning with the quantity and health of the population [16]. 

**Figure 1:**
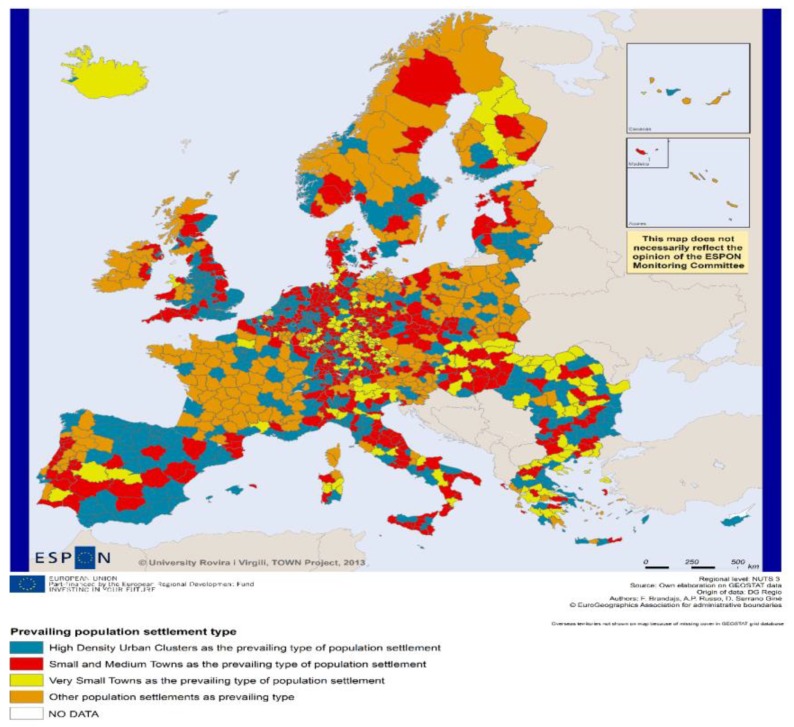
Map typology of demographic status. Source: www.espon.eu [17].

The results of research conducted by the European Union are a rich source of information about the European Union area and offer a diversity of instruments, such as multilevel analysis, typologies, scenarios, and interactive tools, which are useful in spatial planning and territorial development. The demographic changes shown in Figure 1 may be analyzed as an example of the use of a multilevel approach and typology studies. Seven types of region, experiencing many of the various effects of demographic and migration flows, are distinguished here. The maps show that all Lithuanian and Polish regions, as well as other regions of Central and Eastern Europe, have been classified as an area of potential challenges for the workforce. Compared to the EU average, they have a higher proportion of people at a young age. However, the main problem here is a mismatch between the number of economically active people, their education, and aspirations, as well as the opportunities of finding suitable employment in the regional labor market. Therefore, despite the great potential of the quantity of labor, such regions are characterized by a decrease in population, due to lower birth rates and outflows.

Spatial modeling based on demographic projections is burdened with the risk of adopting forecast variants that are too optimistic or pessimistic; therefore, it is essential to verify forecasts annually and to update them. A reliance on long-term forecasts bring with them the potential danger of future economic losses, because the accuracy of any long-term prediction will naturally decrease over time. To counter this, the forecasters would usually prepare various variants of the forecast (minimum, maximum, and intermediate variant), though this solution increases the cost of the studies and makes the process more time-consuming. Ogryzek et al. [18] drew up an algorithm of demographic development for one variant—one that was the most likely and at the lowest risk of using a random factor. He incorporated game theory to obtain a prediction of the future population with the lowest risk of inaccuracy. Game theory is a mathematical theory dealing with the study of optimal behavior in the event of a conflict of interest. It was derived from studies on gambling, and continues to uses that terminology [19]. However, the theory has come to find applications mainly in economics, biology (especially in sociobiology), sociology, and computer science (artificial intelligence). In 2005, Thomas, Schelling, and Aumann received the Nobel Prize in economics for the application of game theory in the social sciences and in microeconomics (for the behavior of individuals and conflict resolution) [20]. The mechanisms used in economic solutions were used as the archetype of mathematical solutions. Ogryzek [18] extended this method to the demographic forecast of a random factor to heighten the element of uncertainty in decision-making events, and his study appears to be accurate in reality-based modeling on an extensive comparative analysis of results from the classical and random factors. His way of solving the problem is to determine the most probable variant projections (preserving the future direction of changes in the basic demographic processes provided by conventional methods), as opposed to an intermediate option between the maximum and minimum variant projections. The algorithm was implemented for the GIS environment using the Python programming language in order to automate the process. Saving time and money when forecasting the most likely future population may streamline the data acquisition process of spatial information systems, improve adjuvant planning processes, and make updating and verification in subsequent years more efficient. In order to verify the utility algorithm, demographic forecasts for Vilnius and Olsztyn from 1997 to 2014 were used.

## 2. Materials and Methods

**Figure 2:**
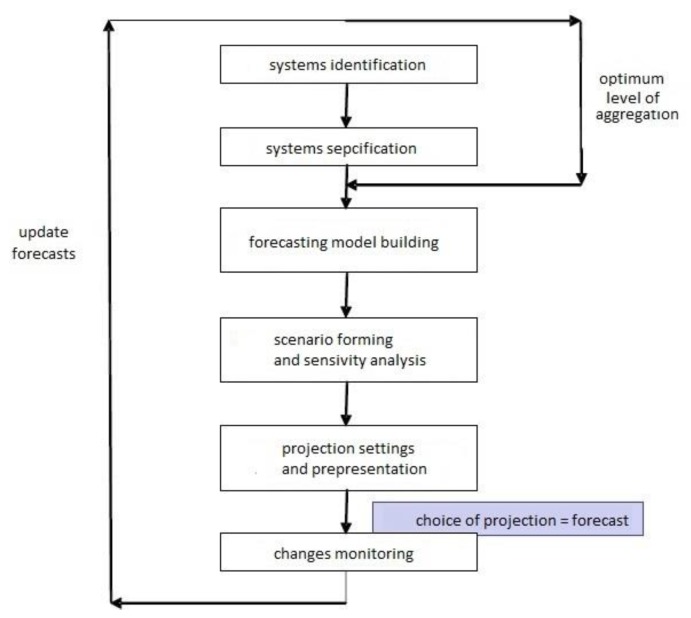
Flowchart of forecasting demographic. Source: [21].

Demographic forecasting looks at the population by age and sex, as well as selected characteristics (e.g., marital status and education), or things like labor resources or the number of households. Forecasting methods can be divided into simple extrapolation methods based on population growth trends or component methods where the population number is equal to the number of births, deaths, and net migration, as well as the cohort-component method. There are also multistate models and stochastic forecasting models. A flowchart of demographic forecasting is shown in Figure 2.

The method of population projection is based on the calculation of successive natural movement and migration based on population numbers and rates by sex and age for subsequent years, resulting in prospective states of population [22]. These calculations are performed at the lowest level of territorial division, with the results for higher levels being obtained by aggregation. The calculations of population size are carried out for the permanently residing population. The total current population—presented as the condition of the population—is obtained by adding the constant balance population in time to the population. The prospective demographic rates for the lowest-level units necessary for calculations are obtained by taking the territorial differentiation of retrospective coefficients and the tendency resulting from prospective national coefficients into account. It is clear that the territorial differentiation factors do not need to persist in the same shape as the baseline in the future. In the case of partial coefficients of births and deaths, the tendency to blur social differences associated with fertility and health trends as a result of the media of mass culture and the growth of social communication, is justified. In the case of internal migration, one can expect dominant trends in favor of more diversified flows, which will be associated with a greater tendency to move without attachment to the place of residence [3].

There are three methods of producing predictive distributions for fertility, mortality, and migration: time-series extrapolation, expert judgement, and an analysis of past forecast errors [4,23]. In practice, a combination of at least two of these methods is used to produce predictive distributions [2]. Cohort-indicator methods are used in forecasts for CSO (GUS) [24], Eurostat [25,26,27,28], the UN [29], with multistate models for Poland being developed by Kotowska [30] and Strzelecki [31], and a stochastic forecast by Matysiak et al. [2]. The demographic forecast by using a random factor, developed by Ogryzek et al. [18], is a modification of the cohort-component method described above, involving the inclusion of mechanisms for solving problems in game theory, with particular emphasis on "playing with nature". Games with nature, according to Trzaskalik [32] and Kukula [33], are double games in which the opponent is nature. The opponent is not interested in the result of the game, so the game is resolved from the point of view of one of the players. The optimal strategy can be achieved using one of the rules of decision-making:

- Wald Criterion (max–min rule)
- Hurwicz Criterion
- Bayesian Criterion
- Optimistic Criterion (max–max rule)
- Savage Criterion (rule of minimum regret).

Getting the most anticipated attributes of natural increase and migration, at their lowest risk of occurrence, will enable the most probable forecast of the future population. The projected population for a given year is the sum of the population from the previous year and the future rate of natural increase and migration.

The state of the population at time t = state population at time t −1 + birth in the period (t − 1, t) − deaths in the period (t − 1, t) + immigration in the period (t − 1, t) − emigration in the period (t 1, t).

L (t) = L (t − 1) + B (t − 1, t) − D (t − 1, t) + I (t − 1, t) − E (t − 1, t)

Aging: L (x + h, s, t + h) = L (x, p, t) p (x, s, t + h)

L (x s, t)—the number of people s sex at the age x at the time t, wherein x = 0, 1, ..., z. 

(source: [34])

Time t is set as the beginning of (1.01) for the period. On this basis, you can determine the population for the next period, and so forth.

p (x, s, t + h) − the probability of living till age x + h year period t + h by a person of a certain gender taken from life table for h = 1 ... n, etc.; the expected value of the parameter index kB, kD, kI, kE (kB—birth rate, kD - death rate, kI—population inflow rate, kE—emigration rate). This indicator is the number of live births, deaths, inflow, and outflow of the population of a thousand people for a given year, and is inversely proportional to the population from the previous year.

B = LD * 1/kB;

D = LD * 1/kD;

I = LD * 1/kI;

E = LD * 1/kE; (own source)

Index k has been determined by computer simulations of the basic demographic processes (rate of natural increase and migration). The simulation performs the search for each attribute in 1000 draws, from 0 to 1000 (singles). Each group of 1000 people plays with population growth and migration, and the prize in the game depends on a number selected at random by the simulator, from 0–1000. If the drawn number is within the confidence interval, a single game is completed successfully (live birth or death, the inflow or outflow of the population). For most of the expected values of the parameter (at the lowest risk), a single game is repeated n times (the number of games is determined by the planner). The value of parameter k is determined by us, using a modification of the formula for the expected value of the game [18].

EV = k_U_ = k_Z_ = k_N_ = k_O_ = (w_1_,w_2_, p_1_, p_2_) = p_1_ w_1_ + p_2_ w_2_ (1)

where:

w_1_, w_2_ May—value parameter

p_1_, p_2_—likelihood parameter

For a more precise measurement of risk, the variances of the game need to be calculated. The greater the deviation from the results, the riskier the game is [35] (Kaminska 2006). The software selects this parameter, that is, the lowest risk, derived from the formula for the variance of the game [18,35].

(2) $$WG=\;\sum_{s\;=\;1}^{n}\mathrm{ps}\;{\left(\mathrm{ws}\;-\;\mathrm{EV}\right)}^{2},$$

where:

w_s_—result of the game

p_s_—probability of occurrence

For each of the attributes, confidence intervals of the parameter were defined (ranges of numbers). These intervals are selected based on mathematical formulas using coefficient attributes (Table 1), where the minimum limit range is the number zero, and the maximum limit used the formula:

Birth
U_97_ = L_96_/W_U97_/1000
U_98_ = L_97_/W_U98_/1000
..................................
U_25_ = L_97_/W_U24_/1000
Deaths
Z_97_ = L_96_/W_Z97_/1000
Z_98_ = L_97_/W_Z98_/1000
…………………….
Z_25_ = L_97_/W_Z24_/1000
Population inflow
N_97_ = L_96_/W_N97_/1000
N_98_ = L_97_/W_N98_/1000
…………………….
N_25_ = L_97_/W_N24_/1000
Emigration
O_97_ = L_96_/W_O97_/1000
O_98_ = L_97_/W_O98_/1000
…………………….
O_25_ = L_97_/W_O24_/1000
L—population, W_U_—birth rate, W_Z_—deaths rate, W_N_—population inflow, W_O_—emigration rate

In order to obtain the most expected value of a given parameter (at its lowest risk), a single game is repeated n times. N in this study was 1000.

The main sources of dates used in the article were: the Central Statistical Office (CSO) and Lietuvos Statistikos Departamente (LSD). Statistics Poland (formerly known in English as the Central Statistical Office) is Poland’s chief government executive agency charged with collecting and publishing statistics related to the country’s economy, population, and society, at the national and local levels. Lietuvos Statistikos Departamente (formerly known in English as Statistics Lithuania), is an institution in Lithuania which is managed by the government. It was founded in 1990 by a resolution during the process of Lithuanian re-establishment of independence, and codified by the Law on Statistics of the Republic of Lithuania. The institution became a member of the European Statistical System in 2004. These are the most reliable statistical institutions that collect and share national data for different purposes (e.g., for EUROSTAT). These data are used for various types of analyses: demographic, sociological, economic, and suchlike. Every formal institution that wants to prepare this type of analysis should use that data.

The source of data for our simulator, obtained from CSO and LSD, were: Olsztyn population in 1996: 168,711 people, Poland in 1999: 38,260 people, and Vilnius population in 1996: 578,327 people, Lithuania in 1999: 3536 people. In the next step, we used rates from Table 1 and repeated the calculations year by year. That way, we could predict the number of people in the following years.

## 3. Results and Discussion

**Figure 3:**
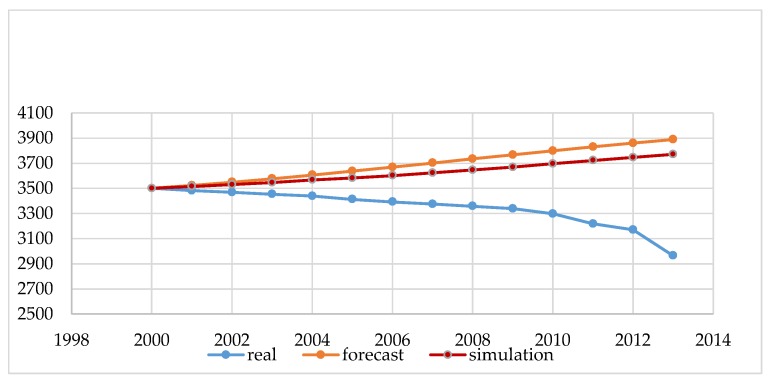
The demographic forecast of Lithuania (population in thousands). Source: own study based on CSO, EUROSTAT, LSD [24,25,26,27,28,36].

**Figure 4:**
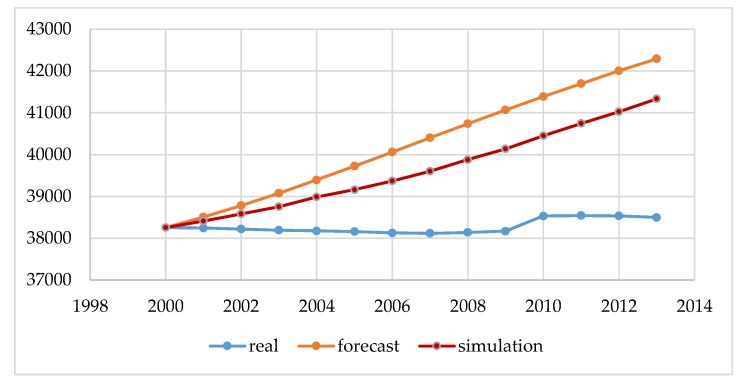
The demographic forecast of Poland (population in thousands). Source: own study based on CSO, EUROSTAT, LSD [24,25,26,27,28,36].

Our preliminary study summarizes demographic data for Poland and Lithuania. Figure 3 and Figure 4 show the real and projected populations by Eurostat, and the result of the estimation made by the authors performed using our simulator according to the methodology described in Section 2: Materials and Methods.

The data shown in Figure 3 and Figure 4 in blue (real) are the actual state from the Statistical Offices, and was not subject to modeling. These are statistical data made available at the request of the governmental institutions both in Poland and in Lithuania. The next element in the chart is the forecast, which is carried out by governmental institutions of Poland and Lithuania for each city above 100,000. Such forecasting should guarantee rational, sustainable development in the area of land development, demography, and spatial planning for a period of 10 to 30 years. These are the legal requirements of forecasts performed in Poland and Lithuania, and the conclusions resulting from them form the basis for all spatial activities, such as changing the form of development from forests into a housing estate with the provision of basic social functions. By analyzing the data from Figure 3 and Figure 4, we can conclude that, for Polish and Lithuania, our original simulation method estimates the future size of the population more accurately. However, decision-making based on either method (prediction and simulation) for such a large area is risky. Our study confirms that the demographic forecasts compiled using a simulation with elements of game theory can help us to predict the population better than by using traditional forecasting methods.

**Figure 5:**
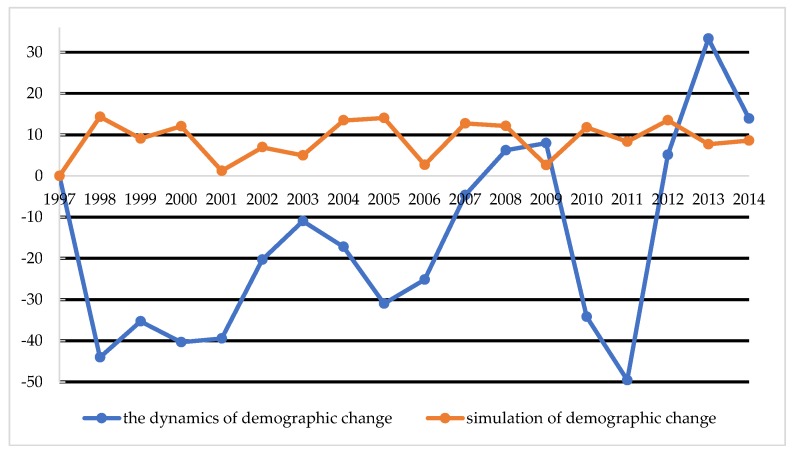
The dynamics of demographic change in Vilnius in the years 1997–2014. Source: own study based on CSO, EUROSTAT, LSD [24,25,26,27,28,36].

**Figure 6:**
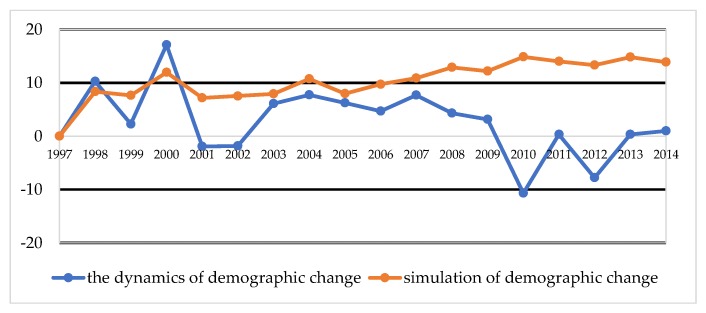
The dynamics of demographic change in Olsztyn in the years 1997–2014. Source: own study based on CSO, EUROSTAT, LSD [24,25,26,27,28,36].

Figure 5 and Figure 6 contain the results of the comparison of demographic changes in Vilnius and Olsztyn in the years 1997–2014, that is, the dynamics of change, with the total amount in thousands.

The dynamics of changes in the population of Vilnius resemble a sinusoid curve that regularly rises and falls, but does not exceed the population of 1997 until after 2008. However, in Olsztyn, with the exception of a slight decline between 2001 and 2002, until 2009 there is generally a positive trend. However, from 2010, strong dynamics of change can be noticed. This situation in Poland happened due to the fact that there was an economic crisis in 2001 and 2002, meaning that the population of Olsztyn migrated to larger cities or emigrated abroad. An important element that was possible to predict was Poland’s accession to the European Union, which brought with it the legal opportunity to work in the EU. A labor outflow of 50,000 people was recorded in Vilnius, and 13,000 people in Olsztyn. The simulated and real data in Figure 5 and Figure 6 show the dynamics of demographic changes from 1997 to 2014. All the abrupt breaks and increases were treated by the forecast simulator as extremes and affected by the smoothing function. It is obvious that, in the simulation process, the authors performed tests using what is known as try n = 1000; that is, the simulation was repeated 1000 times. The larger the sample, the greater the smoothening of results (averaging).

**Figure 7:**
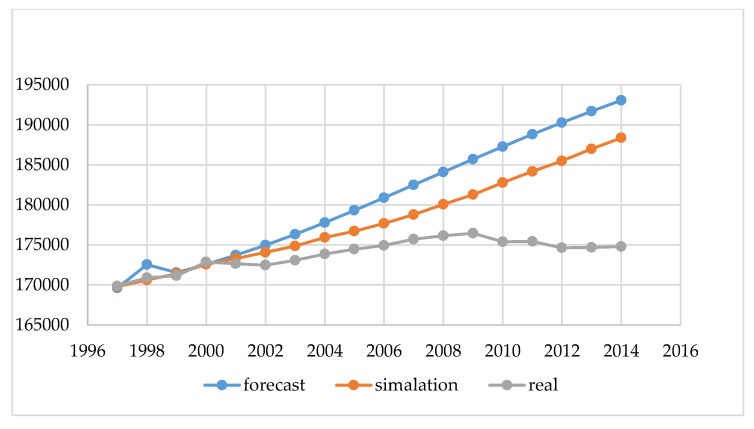
The Demographic Forecast of Olsztyn (Years 1997–2014). Source: own study based on CSO [24].

Figure 7 shows a comparison of: the real population (real), state forecast (forecast), and the simulator (simulation) for Olsztyn in 1997–2014. Similarly to Figure 3 and Figure 4, where the national results were presented, better results were obtained for the city of Olsztyn using our simulator in comparison with state forecasts. In this chart, it is even clearer that forecasting changes in the population for a period longer than five years, regardless of the method used, does not bring satisfactory results. In conclusion, such forecasts should be performed for a maximum period of three to five years.

The use of the simulator requires certain assumptions, and brings with it certain limitations. To start the simulation, you need to have reliable data from public sources indicating the size of the population. It is also necessary to obtain the rates (Table 1) required for calculations from the state statistical institutions. In the absence of such rates, one may attempt to develop it independently, although it would be impossible to compare the obtained results with the state forecasts. The preparing of methodology for calculating such indicators, as well as support of its validity with specific results of demographic forecasts, may be the subject of separate studies. The simulator, just like the state forecasts, is not protected from additional factors which are difficult to predict, which affects changes in the population, such as the economic crisis, major changes in the state policy, and changes in EU membership. Forecasts made by government institutions do not provide different variants of results, but only an optimal size of the population. In the process of the prepared simulation, you can not only increase or decrease the number of repetitions of the forecast (which affects the result), but also increase (or decrease) the share of the random factor. The random factor used in the process of game theory is actually pseudo-random, so the simulator has to develop a certain randomization algorithm based on the assumptions (rates from Table 1). Using the simulator, however, it is possible to treat these assumptions as deterministic or stochastic functions, of which solution (results) does not have to be expected, but simulated in a certain variant. In a stochastic model, we can approximate the appearance of this function to any mathematically known function. This means that the results obtained should be closer to the real values. A limitation of the use of the simulator is also the forecast period, although similar restrictions also apply to state forecasts. The conducted studies indicate a maximum of a three to five year period of the simulator’s applicability, although longer forecasts give better results than state forecasts.

Demographic forecasting can be applied both on the macro scale (countries, regions) and on a local scale (city). When creating the assumptions of the development policy of each country, forecasting of populations is a very important element. Authorities need to know the trends of change in this area in order to properly formulate the social policy of the state (e.g., in the case of negative forecasts, to introduce mechanisms that encourage people to have children; or in the opposite situation, to limit social programs). The results of demographic forecasts also justify the need for development (or lack of development needs) of investments in the field of social and technical infrastructure (health care, education, road networks). Demographic forecasting is even more widely used on a local scale. Local governments, when creating strategies for socio-economic development, need to know the results of demographic forecasts. In this way, the demand (or lack of it) can be determined in terms of specific social and infrastructure investments (e.g., the construction of new housing estates, development of road systems, construction of kindergartens, schools, and healthcare facilities). The city must also predict the relevant needs in the relevant documents, such as concerning new investment areas. In Poland, such planning documents are: a municipal development strategy, a study of the conditions and directions of spatial development of the municipality, and local land-use plans. All these documents form the spatial policy of the municipality in terms of spatial development. Without justifying the investment needs, these documents are not justified. A good research tool that will get the most reliable results of demographic forecasts is indispensable here. Thanks to this, there is a good chance that the development of the city will correspond to the actual needs of its current and future residents.

## 4. Conclusions

The main purpose of the article was to present the author’s method of demographic projections using elements of game theory. The results obtained in this method were compared with the results of the methods currently used for forecasting by the governments of Poland and Lithuania. The obtained results showed that the developed method, based on the same input data and analogous coefficients, gives the possibility of obtaining more probable results. However, the results obtained by taking into account the random factor are more accurate and better than forecasts based on the classic model of reality. The authors noted that such a long forecasting period generates large errors, which is shown in Figure 3 and Figure 4. In connection with the above, erroneous conclusions may be drawn, and thus may disrupt the sustainable development planned for a given city. To improve mathematical modeling, the forecast period should be shortened to a maximum of three to five years. In addition, a study of the distribution of input data should be performed. Thanks to this, it would be possible to get to know the function of the distribution of this data and carry out modeling in three variants: for the average value and for the simulation of the uncertainty value, minimum, and maximum. However, this was not the subject of our research in this article. In future analyses, the authors plan to use this approach.

Additionally, simulations should be preceded by profound expert analysis of demographic change. The choice of factors that characterize the test area should be based on observations of long-term demographic changes. Using the simulator to pick the number of the population is more accurate than according to traditional methods, compared to the real increase in the population. It is necessary to use the same parameters to perform simulations, which are developed according to classical methods. Quantifying the population of cities is a more difficult and complicated process than in small towns. The authors of the article noticed that the use of the forecast method from a developed simulator using game theory by using the same input data models better results, which are closer to reality. However, the method is based on parameters and input data from the Bolesławski method [34] used to predict population numbers in Poland and Lithuania. Therefore, neither of these methods are perfect, though it seems that the method proposed in the article generates better forecast results. In the simulation process, the authors performed tests in the n = 1000 sample. The larger the sample, the greater the smoothening of results (averaging). In the simulator, it is possible to perform tests for a smaller number of attempts, or to indicate the possibility of variants, taking into account only extreme results. In such cases, Figure 5 and Figure 6 would be closer to each other, but such extremes are not easy to predict (e.g., economic breakdowns, stock market crashes, change of currency to the Euro, accession to the EU, and the opening of labor markets) In 1997, such situations in Poland and Lithuania seemed almost unrealistic, as the countries were still operating in different political and economic systems. Nonetheless, while they may not be sufficient by themselves to form the basis of a decision, they can have a role to play in advanced decision-making support, and can provide reliable information about future populations. There is no specified threshold for statistical error that identifies the truth of this hypothesis, though the mutual comparison of the actual population is a reliable source of information about the method.

## Figures and Tables

**Table 1:** List of rates.

Year	Birth	Deaths	Population Inflow	Emigration
1997	1.4	1.18	0.74	0.07
1998	1.4	1.18	0.74	0.07
1999	1.4	1.18	0.74	0.07
2000	1.5	1.18	0.74	0.07
2001	1.5	1.18	0.74	0.07
2002	1.5	1.18	0.74	0.07
2003	1.5	1.18	0.74	0.07
2004	1.5	1.18	0.74	0.07
2005	1.5	1.16	0.74	0.07
2006	1.6	1.16	0.74	0.07
2007	1.6	1.16	0.74	0.07
2008	1.6	1.16	0.85	0.07
2009	1.6	1.16	0.95	0.07
2010	1.6	1.16	1.05	0.07
2011	1.58	1.16	1.15	0.07
2012	1.58	1.16	1.15	0.07
2013	1.58	1.16	1.15	0.07
2014	1.58	1.16	1.15	0.07
2015	1.58	1.14	1.15	0.07
2016	1.58	1.14	1.15	0.07
2017	1.58	1.14	1.15	0.07
2018	1.58	1.14	1.15	0.07
2019	1.58	1.14	1.15	0.07
2020	1.58	1.14	1.15	0.07
2021	1.58	1.14	1.16	0.07
2022	1.58	1.14	1.17	0.07
2023	1.58	1.14	1.18	0.07
2024	1.58	1.14	1.19	0.07
2025	1.58	1.12	1.2	0.07

Source: [34].
